# Advances in the Study of Pluripotent Stem Cells in Livestock

**DOI:** 10.1111/cpr.70008

**Published:** 2025-02-24

**Authors:** Xinyi Zhou, Chen Gao, Wenxuan Zhao, Xinhua Wei, Dawei Yu, Huiying Zou, Weihua Du

**Affiliations:** ^1^ Institute of Animal Science Chinese Academy of Agricultural Sciences Beijing China

**Keywords:** application prospects, embryonic stem cells, induced pluripotent stem cells, livestock

## Abstract

Livestock pluripotent stem cells, derived either from early embryos or induced through somatic cell reprogramming technology, possess the unique ability to self‐renew, maintain an undifferentiated state and differentiate into various cell types. Consequently, the generation of PSCs from agricultural animal species holds great potential for applications in livestock breed improvement, rapid propagation, disease modelling and xenotransplantation. However, compared to the great achievements made in mouse and human pluripotent stem cells research, the generation of livestock pluripotent stem cells still remains challenging. This article offers an overview of the classification, regulatory mechanisms of pluripotency, and developmental history of livestock pluripotent stem cells, while also anticipating their future application prospects. These insights provide valuable references for the reproduction and breeding of large livestock.

## Graphical abstract

Livestock pluripotent stem cells include embryonic stem cells (ESCs) isolated from the inner cell mass of blastocysts and induced pluripotent stem cells (iPSCs) derived from somatic cell reprogramming. They hold significant potential for applications in livestock breed improvement, rapid propagation, human disease modelling and human organ transplantation.

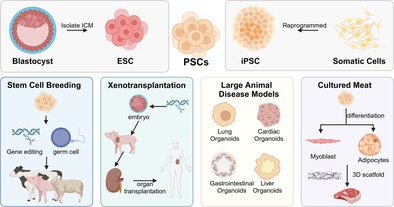

## 1 Introduction

**FIGURE 1:**
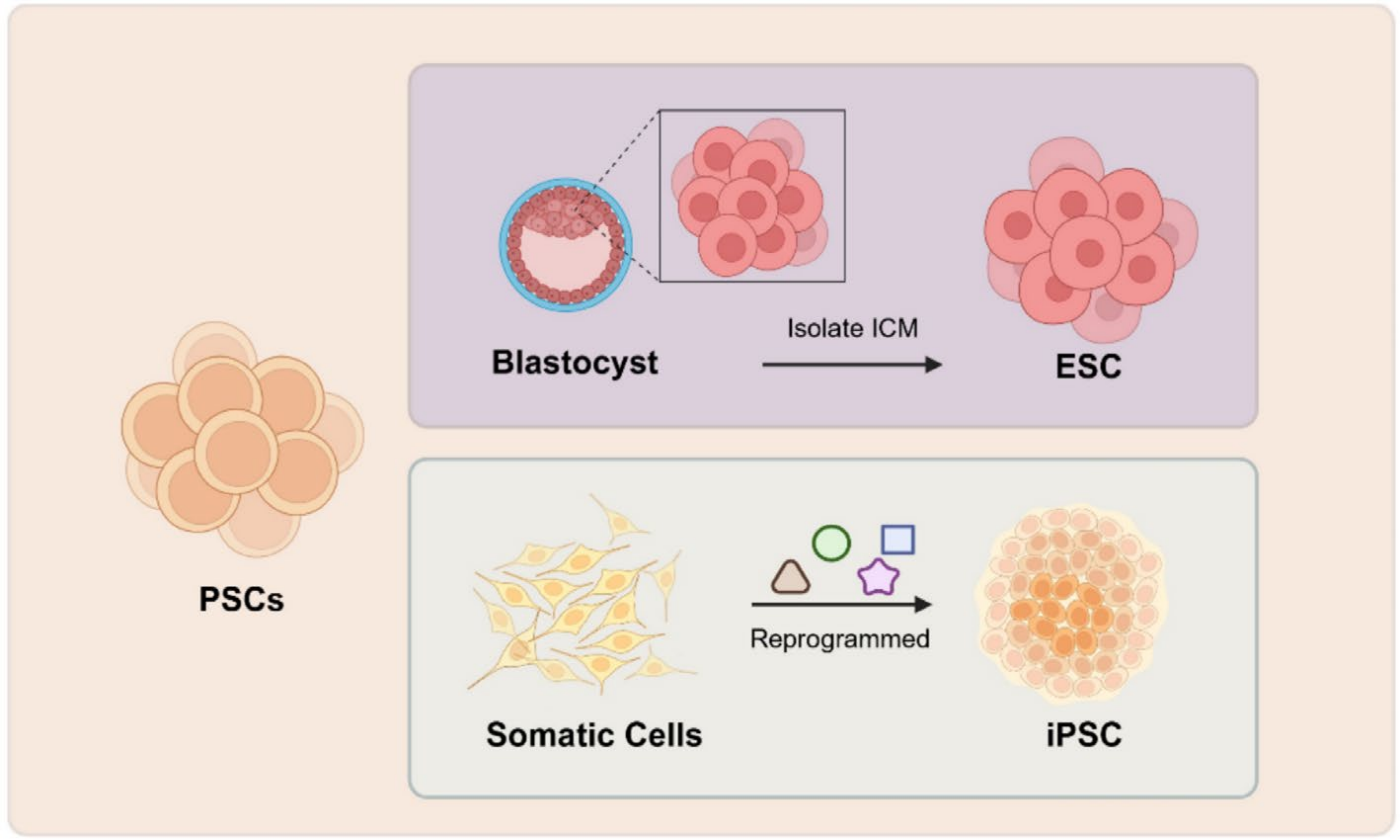
Type of pluripotent stem cells in livestock. PSCs include ESCs and iPSCs; ESCs are a type of cell isolated from early embryos (before the gastrula stage) or primordial gonads. iPSCs refer to a class of stem cell lines that can proliferate indefinitely, self‐renew, and differentiate into various cells of the three germ layers, generated by overexpressing specific transcription factors to reprogram terminally differentiated somatic cells.

Stem cells are a class of cells derived from animal embryos or adult tissues that have the ability to continue self‐renewal and differentiate into any cell type of the three germ layers. Based on their differentiation potential, stem cells can be classified into totipotent stem cells, pluripotent stem cells (PSCs), multipotent stem cells and unipotent stem cells [1]. PSCs include embryonic stem cells (ESCs) isolated from the inner cell mass (ICM) of blastocysts and induced pluripotent stem cells (iPSCs) derived from somatic cell reprogramming; both possess multidirectional differentiation and self‐renewal capabilities (Figure 1) [2]. With the integration of modern life science technologies such as gene editing, somatic cell cloning, single‐cell sequencing and in vitro embryo production, PSCs have shown immense potential in applications including livestock breed improvement and rapid propagation, germplasm resource conservation, large animal disease model creation and xenotransplantation.

Significant breakthroughs have been achieved in the research of rodent and human PSCs, particularly mouse ESCs and iPSCs. Mouse ESCs and iPSCs have been shown to pose germline transmission potential and have successfully produced germline chimeras. Moreover, mouse offspring entirely derived from ESCs or iPSCs have been generated using tetraploid complementation technology. However, progress in PSC research for livestock such as pigs, cattle and sheep has been slow. Although morphologically similar livestock PSCs capable of differentiating into the three germ layers and forming teratomas have been obtained, they do not have the ability to be transmitted by germline. This article summarises the classification, regulatory mechanisms of pluripotency, and developmental history of livestock PSCs, and anticipates their application prospects in various fields, including livestock breed improvement, which would provide a theoretical reference for the successful establishment of high‐quality PSCs with germline chimerism capabilities.

## 2 Types of Livestock PSCs and Their Basic Characteristics

**FIGURE 2:**
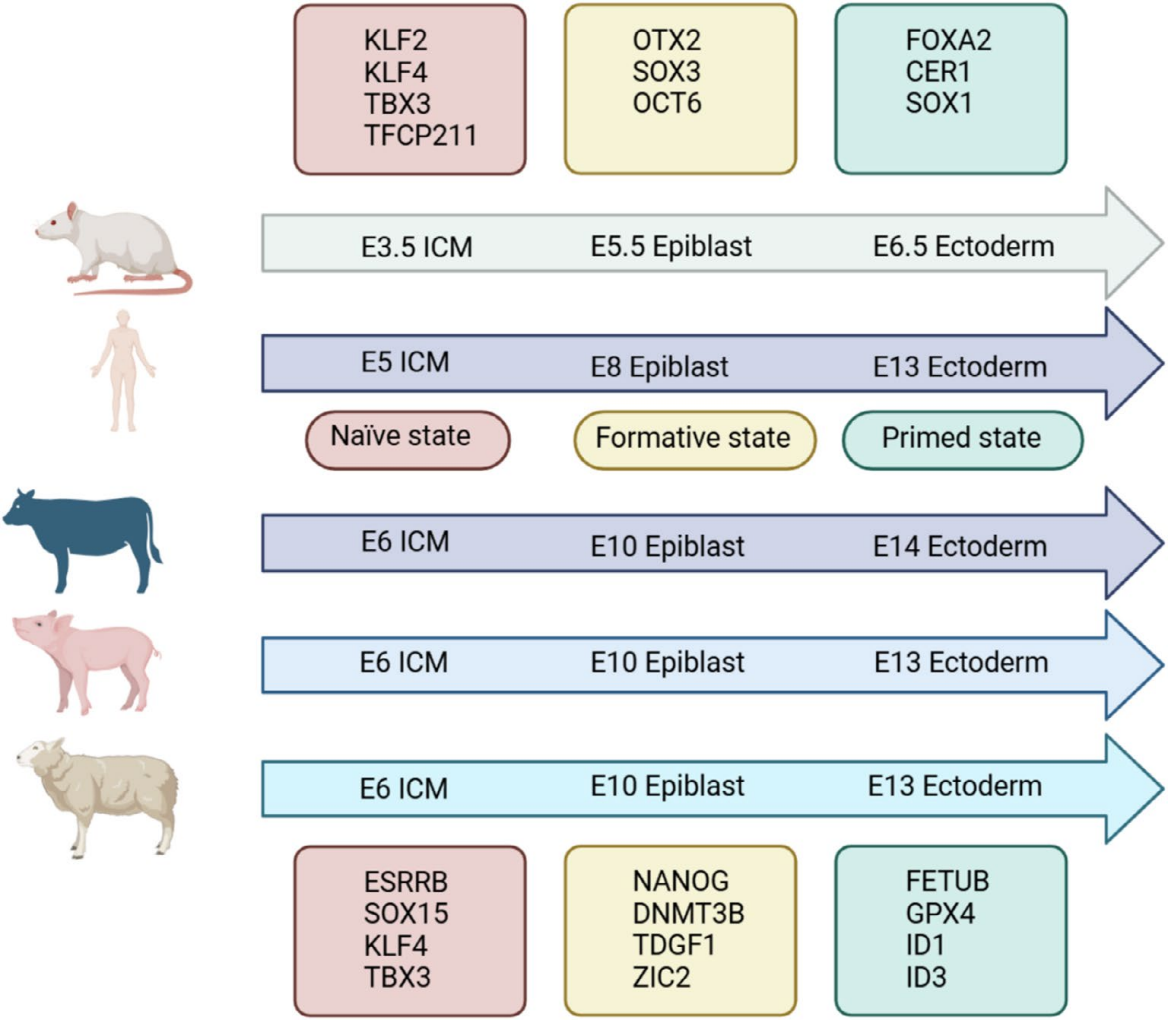
Pluripotent state of embryonic stem cells in livestock. ESCs can be classified into three states based on differences in isolation timing and pluripotency status: Naïve, Formative and Primed. For mouse, the Naïve ESC was isolated from the ICM of E3.5. Formative ESCs were isolated from E5.5 epiblast. The Primed state is isolated from E6.5 Ectoderm. For human, the Naïve ESC was isolated from the ICM of E5. Formative ESCs were isolated from E8 epiblast. The Primed state is isolated from E13 Ectoderm. For livestock, the Naïve ESC was isolated from the ICM of E6. Formative ESCs were isolated from E10 epiblast. The Primed state is isolated from E14 or E13 Ectoderm.

ESCs are a type of cell isolated from early embryos (before the gastrula stage) or primordial gonads. They are characterised by their ability to proliferate indefinitely in vitro, self‐renewal and differentiate into multiple lineages. Taking mice as an example, ESCs can be classified into three states based on differences in isolation timing and pluripotency status: Naïve, Formative and Primed. Naïve state ESCs, derived from ICM of pre‐implantation blastocysts, exhibit a tightly packed dome‐shaped colony morphology with high cloning efficiency. Female Naïve state cells possess two active X chromosomes (XaXa), and can express pluripotency factors such as OCT4, NANOG, SOX2, KLF2, KLF4, and Naïve‐specific markers including REX1, NROB1 and FGF4. When cultured in vivo, Naïve state cells can differentiate into all somatic and germline lineages and are capable of forming somatic chimeras and germline chimeras. Formative state ESCs exist between the Naïve and Primed pluripotency states, originating from the epiblast layer after implantation and before gastrulation. They express specific markers such as TUJ1, SOX17, CTNT and FOXA2 and can directly respond to germ cell induction signals, colonising somatic tissues and reproductive lineages in chimeras [3]. Primed state ESC soriginate from the epiblast layer of post‐implantation embryos. They exhibit a loose and flattened colony morphology with low cloning efficiency. The female cells have one active and one inactive chromosome, exhibiting an XaXi status. These cells express pluripotency factors such as OCT4, NANOG and SOX2, along with Primed‐specific markers like FGF5. Primed state ESCs exhibit biased differentiation and can form teratomas but lack the capacity to form chimeras. During mouse embryo implantation, the blastocyst ICM differentiates into the epiblast and hypoblast [4]. The establishment of livestock ESCs was consistent with that of mouse ESCs except for the different isolation time and expression factors. For livestock, the Naïve ESC was isolated from the ICM of E6 and could express the Naïve pluripotency factors ESRRB, SOX17, KLF4, TBX3, etc. Formative ESCs were isolated from E10 epiblast and expressed NANOG, DNMT3B, TDGF1, ZIC2, etc. The Primed state is isolated from E14 or E13 Ectoderm and can express FETUB, GPX4, ID1, ID3, etc (Figure 2) [5]. Additionally, the regulatory mechanisms of pluripotency in livestock ESCs t are not entirely identical to those in mice, although OCT4, SOX2 and NANOG are important transcription factors maintaining their pluripotency [2].

iPSCs refer to a class of stem cell lines that can proliferate indefinitely, self‐renew and differentiate into various cells of the three germ layers, generated by overexpressing specific transcription factors to reprogram terminally differentiated somatic cells [6]. Similar to ESCs, iPSCs are round with large nucleoli, scant cytoplasm, and have a tightly packed dome‐shaped colony morphology. Also, the gene and protein expression, as well as epigenetic modification patterns of iPSCs are highly similar to those of ESCs, and they also possess similar differentiation capabilities, being able to achieve trilineage differentiation both in vitro and in vivo. Most importantly, iPSCs have diploid chimerism capabilities, and viable offspring can be obtained through tetraploid complementation. Moreover, multiple rounds of gene editing on iPSCs do not affect their differentiation and expansion capabilities. Finally, iPSCs can be produced on a large scale in the laboratory without the need to breed live animals; they are directly derived from tissue cells such as skin, blood and fat, without the need to destroy embryos or eggs, thus avoiding ethical controversies.

## 3 Mechanism of Pluripotency Regulation of Livestock PSCs

Livestock PSCs can proliferate indefinitely and possess pluripotent developmental potential, while the regulation of their pluripotency is a complex process involving the interaction of multiple signalling pathways and transcription factors. The combined action of these mechanisms maintains the pluripotent state of stem cells.

### 3.1 Regulation of PSC Pluripotency by Different Signalling Pathways

Research indicates that multiple signalling pathways are involved in the maintenance and regulation of the pluripotent state of PSCs, including WNT/β‐catenin, JAK/STAT3, FGF/ERK, Activin/Nodal and BMP, among others. The WNT/β‐catenin signalling pathway plays a crucial role in embryonic development, stem cell proliferation and somatic cell reprogramming [7]. Activating this pathway can enhance the self‐renewal capacity of stem cells and the expression of pluripotency genes, as well as control stem cell differentiation [8]. Glycogen synthase kinase (GSK) 3 is a key regulator of the WNT signalling pathway. GSK can phosphorylate β‐catenin and degrade it in a directed manner, thereby inhibiting the WNT signalling pathway. CHIR99021 is one of the inhibitors of GSK3, which activates the WNT pathway by inhibiting GSK3, promoting stem cell self‐renewal [9]. When CHIR99021 and leukaemia inhibitory factor (LIF) are added to the culture medium of bovine iPSCs, the cell clones become tightly packed and stable across passages [10]. Regulating the WNT/β‐catenin signal transduction in porcine PSCs can prevent their differentiation into mesoderm [11].

The JAK/STAT3 signalling pathway is associated with cell proliferation, differentiation, apoptosis and immune regulation [12], but it is not essential for the establishment of pluripotency in stem cells. The IL‐6 family is the main activation signal of JAK/STAT3, and LIF is a member of the IL‐6 family. Activation of the JAK/STAT3 signalling pathway by adding LIF is crucial for maintaining the pluripotency of stem cells [13]. Additionally, transcription factors KLF4, GBX2 and TFCP2L1 are also associated with the STAT3 activation. Activation of the JAK/STAT3 signalling pathway can maintain the self‐renewal of mouse ESCs in a pluripotent state and is involved in the formation of pluripotent cells in pre‐implantation porcine embryos [14].

The FGF/ERK signalling pathway plays distinguished roles in numerous cellular functions, including embryonic development, tissue regeneration and cancer. FGF4/ERK signalling promotes the differentiation of PSCs and is inhibited during the maintenance of embryonic stem cells and the development of pre‐implantation embryos [15, 16]. The fibroblast growth factor (FGF) family is comprised of 22 ligands that interact with four FGF receptors, regulating fundamental cellular processes such as embryonic development and homeostasis. Extracellular signal‐regulated kinase 1 (ERK1) and ERK2 are two important members of the mitogen‐activated protein kinase (MAPK) family. Activation of FGF can trigger the MAPK pathway, leading to the phosphorylation of ERK1 and ERK2; subsequently, they translocate to the nucleus, activating downstream genes, including NANOG, promoting the maintenance of cell pluripotency. PD0325901 is an inhibitor of MEK, a key enzyme in the MAPK pathway. Inhibition of MAPK signal transduction is crucial for regulating stem cell pluripotency. All cells in the ICM transmit signals through the FGF/ERK signalling pathway with varying durations and amplitudes. Primitive endoderm cells exhibit higher ERK activity, while pluripotent epiblast cells show lower basal activity [17, 18].

The Activin/Nodal and BMP signalling pathways play crucial roles in the proliferation and differentiation of stem cells, determining their fate. Activin A, Nodal, and bone morphogenetic protein (BMP) are members of the transforming growth factor‐beta superfamily [19]. Research indicates that both Activin/Nodal and FGF2 signalling pathways are essential for maintaining the self‐renewal of human embryonic stem cells (ESCs) [20]. Activin induces the phosphorylation of SMAD2/3 proteins, enabling them to interact with downstream transcription factors OCT4 and NANOG. This interaction orchestrates the upregulation of pluripotency‐associated genes like SOX2 and inhibits the differentiation of stem cells into mesodermal and trophoblastic lineages [21]. Controlling the Activin/Nodal signal transduction pathway in human pluripotent stem cells leads to reduced expression of differentiation‐related genes and results in stem cells with enhanced metabolic activity [22]. Early activation of Activin signalling can disturb the equilibrium between ICM and trophectoderm (TE), hindering the derivation of mouse pluripotent stem cells (PSCs) from pre‐implantation embryos [23].

### 3.2 Regulation of iPSC Pluripotency by Reprogramming Factors

Initially, Yamanaka and colleagues identified OCT4, SOX2, KLF4 and c‐Myc (collectively termed the OSKM factors) from 24 reprogramming factors by using Fbx15 as a core gene. The introduction of four factors by viral transduction can induce reprogramming of mouse fibroblasts into induced pluripotent stem cells (iPSCs) [24]. Building on this, replacing the OSKM with other factors also successfully generated mouse iPSCs, such as substituting SOX1 or SOX3 for SOX2, KLF2 for KLF4, and L‐Myc or N‐Myc for c‐Myc [25]. However, attempts to replace OCT4 with its family members OCT1 and OCT6 were unsuccessful in inducing iPSCs, thus confirming that OCT4 is considered the most critical reprogramming factor. Additionally, NANOG and LIN28 can effectively replace c‐Myc and KLF4 to reprogram human umbilical cord mesenchymal stem cells into iPSCs. Simultaneously, the regulation of the LIN41 and WNT pathways can significantly increase the reprogramming efficiency from 0.0004% to 2% [26]. Yu et al. successfully induced human fetal fibroblasts into iPSCs using a combination of OCT4, SOX2, NANOG and LIN28, achieving a reprogramming efficiency close to that of the OSKM combination [27].

Compared to the classic OSKM four factors, removing one or several factors and relying solely on the endogenous expression of the cells can reduce reprogramming efficiency. For instance, a three‐factor induction method without c‐Myc, using only OCT4, SOX2 and KLF4, successfully established mouse iPSCs capable of germline transmission and avoided the potential oncogenic risk associated with the proto‐oncogene c‐Myc [28, 29]. It is evident that c‐Myc is not an essential factor for reprogramming, but it does affect the efficiency of reprogramming. Additionally, using only SOX2, c‐Myc and KLF4 can also generate iPSCs with developmental potential, but the induction efficiency is 30% lower than that of the OSKM four‐factor combination [30].

By expanding upon the foundational OKSM quartet with four supplementary elements—NANOG, hLIN28, hRAG and hLRH1—an augmented eight‐factor regimen has been devised that facilitates swift and proficient conversion of human cells into iPSCs [31], mirroring the phenotypic traits of their OSKM‐derived counterparts such as pluripotency marker expression, teratomagenesis and chimera formation. Predominantly, this enhanced eight‐factor framework is harnessed for generating iPSCs in larger species, where the incorporation of human pluripotency genes markedly amplifies the reprogramming efficacy [32]. Moreover, using episomal vectors to deliver these eight factors during fibroblast reprogramming offers a transient solution, with vectors progressively fading during subsequent cultivation and subculture, thereby fostering iPS lines devoid of persistent exogenous gene sequences [33].

## 4 Development History of Livestock ESCs

The groundbreaking work of Evans and Martin in 1981 marked the first successful isolation of ESCs from the ICM of murine blastocysts, leading to the establishment of viable cell lines outside the body [30]. Throughout the ensuing decade, similar endeavours in livestock species—including pigs, cattle and sheep—yielded ES‐like cell cultures. However, these early efforts encountered challenges such as restricted proliferative capacity (low passage numbers) and constrained developmental versatility (limited differentiation potential). A pivotal shift occurred in the early years of the 21st century when, for the first time, researchers managed to isolate bona fide livestock ESCs exhibiting a ‘Primed’ pluripotency state, thereby overcoming previous limitations.

### 4.1 Development History of Porcine ESCs

Due to the physiology, anatomy, immunology and genomic similarities between pigs and humans, pigs are particularly well‐suited as models to explore human growth and pathology [34]. Scientists have been experimenting with the culture of porcine embryonic stem cells (ES) since the 1990s. In 2019, a milestone breakthrough was achieved in pig stem cells when Pengtao Liu's team obtained the first expanded pluripotent stem cells (EPSCs) from Day 5 pig embryos. These cells demonstrated unparalleled proliferative capacity and multilineage differentiation capabilities [31]. Building upon this success, the group had elucidated key molecular mechanisms governing cellular fate decisions and pioneered techniques for harvesting stem cells from nascent developmental stages—the four‐cell or eight‐cell embryo—thereby paving the way for the derivation of murine EPSC counterparts [35]. The pEPSCM cultivation platform has enabled the derivation of porcine EPSCs from individual blastomeres, with these cells exhibiting the capacity to differentiate along three distinct embryonic lineages: ESCs, placental cells and endodermal derivatives akin to the yolk sac. These pluripotent cells express classic pluripotency markers, such as OCT4, SOX2 and NANOG, and are capable of triger layer tissue differentiation in vivo and in vitro. Notably, they also demonstrate the ability to generate PGC‐like progeny under in vitro conditions. In a parallel breakthrough, Han Jianyong's research group has harnessed an innovative 3i/LAF cocktail comprising CHIR99021, IWR‐1, WH‐4‐023 and LIF to propagate pgEpiSCs—pregastrulated epiblast stem cells extracted at developmental stages spanning E8 to E10. These pgEpiSCs have exceptional proliferative capacity and have undergone more than 260 subculture iterations. The team performed three rounds of gene editing on the obtained pgEpiSCs, then transplanted the edited cells as nuclear donors, and finally produced genetically engineered cloned pigs with multiple modifications [36].

### 4.2 Development History of Bovine ESCs

As a significant economic animal, research on bovine embryonic stem cells (ESCs) is crucial for enhancing livestock production efficiency and quality as well as for the development of new drugs and therapeutic methods in the medical field. Initially, using mouse ESC culture conditions ([MEM]‐Alpha medium was supplemented with fetal calf serum [FCS], mercaptoethanol, glucose, HEPES‐buffer, LIF), researchers successfully isolated and cultured bovine ESC‐like cells from the ICM of bovine blastocysts, but these cells proliferated only up to the fourth passage. Additionally, bovine ESC‐like cells were obtained using human ESC culture conditions supplemented with basic fibroblast growth factor (bFGF) 80% knockout DMEM (KO‐DMEM), 20% FBS, glutamine, β‐mercaptoethanol, 1% NEAA, LIF, FGF, yet these cells could not self‐renew and spontaneously differentiated [37]. Therefore, using mouse or human ESC culture conditions failed to establish true bovine ES cells [38]. Pluripotent bovine ESCs (bESCs) were efficiently obtained from bovine blastocysts by the application of a new CTFR medium, addition of FGF1 and IWR2 factors to the basal ESC medium. These cells exhibit stable morphology, transcriptome profiles, karyotypes, population doubling times, expression of pluripotency marker genes and epigenetic characteristics. Furthermore, they can serve as nuclear transfer donors, yielding normal blastocyst rates. This advancement opens up new possibilities for genome selection, genome editing, and the production of high‐genetic‐value cattle [39]. By further optimising the culture system with the addition of NBFR (N2B27 and 1% BSA) to the CTFR culture base, NBFR‐bESCs were established. These cell lines can be passaged up to 35 times, express pluripotency genes, maintain a normal karyotype, and possess the potential to differentiate into all three germ layers [40]. In the same year, bovine EPSCs were also established. After gene editing, these cells can be used as donors for somatic cell nuclear transfer. This study reported key techniques for inducing bovine EPSCs without reliance on exogenous genes and characterised their biological features, marking a milestone achievement in the research of herbivorous livestock stem cells [32].

### 4.3 Development History of Sheep ESCs

Sheep are important ruminants with a wide distribution and large population. However, research on sheep ESCs has been limited due to the lack of research on the embryonic development process and related signalling pathways in sheep. Pawar et al. isolated the inner cell mass from in vitro fertilised goat blastocysts and co‐cultured it with goat oviductal epithelial cells to establish ESC‐like cells expressing alkaline phosphatase and Oct4 positively [41]. Subsequently, ESC‐like cells were derived from mechanically isolated inner cell masses of in vitro cultured goat blastocysts, exhibiting normal morphology, karyotype, and positive alkaline phosphatase staining, as well as expressing OCT4, SOX2 and Nanog [42]. In the same year, Huang Juncheng's laboratory successfully isolated sheep ESCs from sheep embryos. These cells could be cultured on a feeder‐free medium containing N2, B27, GSK3 inhibitor (CHIR99021) and bFGF. In 2020, Wu Jun's laboratory isolated ESCs from sheep blastocysts that could be passaged up to 40 times. These cells were cultured in a chemically defined system with the addition of fibroblast growth factor 2 (FGF2) and Wnt inhibitor (IWR1). They could form teratomas with all three germ layers in vitro but were unable to form chimeras [43].

The analytical descriptions for each of the ESCs discussed are detailed in Table 1.

**TABLE 1:** The history of ESCs.

Cell type	Initiation cells	Cultivation conditions	Characteristics	Reference
Pig pluripotential embryonic cell	E6–7 pig blastocysts	DMEM; FBS; L Glu; 2ME; P/S	Prone to spontaneous differentiation; Prone to spontaneous differentiation	Maintenance and differentiation in culture of pluripotential embryonic cell lines from pig blastocysts
Pig expanded potential stem cells	E5 blastocysts	pEPSCM: N2B27; DMEM/F 12; NEAA; L‐Glu; 2ME; P/S; CHIR; WH‐4‐023; XAV939 or IWR‐1‐endo; Vc; LIF; Activin A, FBS	Stable morphology; < P40, reprogramming efficiency 10%; normal karyotypes	Establishment of porcine and human expanded potential stem cells
PIG pregastrulation epiblast stem cells (pgEpiSCs)	E8–E10 pig pregastrulation epiblast	3i/LAF: WNTi, GSKi, SRCi, LIF, Activin A, FGF2	> P200, three times gene editing, through nuclear transfer technology, cloning pigs	Generation and characterisation of stable pig pregastrulation epiblast stem cell lines
Bovine embryonic stem cell‐like cell lines	ICM from Bovine demi‐blastocysts	(MEM)‐Alpha, fetal calf serum (FCS), mercaptoethanol, glucose, HEPES‐buffer, LIF	Grew considerably slower than murine ES cells, < P4	Bovine embryonic stem cell‐like cell lines cultured over several passages
Bovine ES cell line	NT, IVF and parthenogenetically activated bovine embryos	80% knockout DMEM (KO‐DMEM), 20% FBS, glutamine, β‐mercaptoethanol, 1% NEAA, LIF, FGF2	Failed to self‐renew and became spontaneously differentiated; pluripotent bovine ES cells with morphology similar to those of established ES cells in humans and mice as well as marker‐staining patterns identical to those of the bovine blastocysts	Generation and characterisation of pluripotent stem cells from cloned bovine embryos
CDX2‐KD bESCs	E6–E7 ICM from SCNT blastocysts	KO‐DMEM, FGF1, IWR2, Glutamine, NEAA, bFGF, hLIF, β‐mercaptoethanol, FBS	With partial characters of pluripotent stem cells, which were still short of capcity to generate chimeras and germ‐line transmission	Establishment of bovine embryonic stem cells after knockdown of CDX2
CTFR‐bESCs	The IVM‐IVF embryos	CTFR: mTeSR1, hFGF2, IWR1	Table morphology, transcriptome, karyotype, population‐doubling time, pluripotency marker gene expression, and epigenetic features. When used as donors for nuclear transfer, bESCs produced normal blastocyst rates	Efficient derivation of stable primed pluripotent embryonic stem cells from bovine blastocysts
NBFR‐bESCs	Zona‐free blastocysts or isolated ICMs	NBFR: N2B27, 1%BSA, IWR‐1, CHIR99021, Activin A, FGF2, TGFβ1	Pluripotent marker expression; normal karyotypes; > P35; formed embryoid bodies in vitro and teratomas in vivo	Simplification of culture conditions and feeder‐free expansion of bovine embryonic stem cells
bEPSCs	Bovine preimplantation embryos	bEPSCM: mTeSR12‐mercaptoethanol, CHIR99021, WH‐4‐023, XAV939, IWR‐1, Vitamin C, LIF, Activin A	These stem cells propagate robustly in long‐term culture, permit precise genome editing, and generate both embryonic and extraembryonic cell lineages in vitro and in chimeras	Establishment of bovine expanded potential stem cells
bEpiSCs	E7, E10, E12 and E14 bovine embryonic	3i/LAF: GSK3β inhibitor CHIR99021, SRC inhibitor WH‐4‐023, WNT inhibitor IWR‐1‐endo, LIF, Activin A and FGF2	Formative EpiSCs	Elucidation of the pluripotent potential of bovine embryonic lineages facilitates the establishment of formative stem cell lines
Goat stem cell‐like outgrowths	In vitro fertilised goat blastocysts	DMEM, FCS, NEAA, β‐mercaptoethanol, mLIF, L‐glutamine	Oct‐4 expression, expression of alkaline phosphatase	Stem cell‐like outgrowths from in vitro fertilised goat blastocysts
Goat stem cell‐like cells	ICMs were isolated from expanded and hatched blastocysts	DMEM, FCS, mLIF, NEAA, β‐mercaptoethanol, L‐glutamine	Stem cell like morphological features, normal karyotype, and expressed stem cell specific markers	Isolation and characterisation of embryonic stem cell‐like cells from in vitro produced goat (*Capra hircus*) Embryos
Ovine embryonic stem‐like cell lines	E6–E8 ICM	N2B27, LIF, BMP4, CHIR99021, bFGF	Stable morphology; positive AP staining; expression of pluripotency genes Oct‐4, Sox2, Nanog; > P30	Derivation and characterisation of ovine embryonic stem‐like cell lines in semi‐defined medium without feeder cells
Sheep embryonic stem cells	Sheep blastocysts	Chemically defined culture system for FGF2, Wnti (IWR1).	Stable morphology; pluripotent marker expression; normal karyotypes; > P40	Derivation of sheep embryonic stem cells under optimised conditions

## 5 Establishment of Livestock iPSCs

Since Yamanaka and colleagues first successfully induced mouse fibroblasts into iPSCs using the classic four factors OSKM, similar approaches have been applied to establish iPSC lines from livestock such as pigs, cattle and sheep.

### 5.1 Development History of Porcine iPSCs

After introducing the human OSKM factors into domestic pig and Tibetan pig fetal fibroblasts, both domestic pig and Tibetan pig iPSCs were successfully obtained. These cell lines not only exhibited ES‐like morphology but also had a normal karyotype, positive alkaline phosphatase staining, and could differentiate into teratomas with all three germ layers, although they could not form chimeras [44]. However, by adding LIN28 to the human OSKM factors, porcine fetal fibroblasts could be induced into iPSCs that specifically differentiated into spontaneously beating cardiomyocytes. Other culture systems have also been used to obtain porcine iPSCs, such as the LBX culture system, which can reprogram porcine fetal fibroblasts into mouse ESC‐like porcine iPSCs with two active X chromosomes [45]. In 2023, Han Jianyong's team used the 3i/LAF culture system to induce high‐quality, integration‐free iPSCs from fibroblasts of aged and rare local pigs. Moreover, after gene editing, using these cells as nuclear transfer donors, the constructed embryos could develop in vitro to the blastocyst stage, demonstrating the potential to produce nuclear transfer individuals [33].

### 5.2 Development History of Bovine iPSCs

In 2011, bovine iPSC‐like cells were successfully established for the first time from bovine fetal fibroblasts, but the reprogramming was incomplete and self‐renewal capacity was limited [46]. Subsequently, transposon induction systems like *PiggyBac* and Sleeping Beauty were used in bovine iPSC research, establishing bovine iPSCs that could be stably passaged for over 50 generations with a monoclonal formation efficiency of 40% [47]. Using the classic OSKM factors and a naïve culture system (naïve medium [NM]), testicular supporting cells were induced into bovine iPSCs exhibiting human iPSC‐like morphology, which maintained pluripotency in vitro and in vivo even after multiple rounds of cryopreservation and thawing [48]. Additionally, using bovine fetal fibroblasts as feeder layers instead of mouse fetal fibroblasts resulted in the establishment of more completely reprogrammed bovine naive iPSCs [49].

### 5.3 Development History of Sheep iPSCs

Using the classic four factors OSKM along with LIN28, NANOG, SV40 and hTERT (OSKMNLST)induction system, the world's first sheep iPSCs were obtained. These cell lines exhibited ES‐like characteristics, including morphology, AP activity, expression of ESC pluripotent genes, and the ability to differentiate into three germ layers in vitro and in vivo [50]. However, sheep iPSCs could also be established using only the OSKM factors. When injected into diploid and tetraploid blastocysts, they formed chimeric ICMs [51]. Injection of sheep iPSCs into fertilised eggs, 8‐cell stage embryos, or blastocysts followed by in vitro culture to the blastocyst stage and transplantation into surrogate ewes resulted in live lambs, confirming the totipotency of sheep iPSCs [52]. Subsequently, a bFGF and activin A‐dependent sheep iPSC line was established; however, it exhibited abnormal sex chromosomes, and endogenous NANOG expression was not activated. Using sheep iPSCs as a nuclear donor, transgenic expression persisted in nuclear transfer embryos, but reprogramming was abnormal. After introducing the OSKMNLST eight factors into sheep kidney cells using a tetracycline lentivirus system, it was found that overexpression of miR‐200c‐141 could improve the epithelial‐mesenchymal transition process by regulating the TGF‐β signalling pathway, enhancing the efficiency of sheep somatic cell reprogramming [53]. Similarly, using the piggyBac transposon system carrying OSKMNLST eight factors, sheep iPSCs expressing DOX‐inducible transcription factors were obtained. These cells could form chimeras when injected into early sheep and mouse blastocysts or cultured in vitro for 6.5 days in mouse embryos, demonstrating stable morphology, in vitro differentiation potential, and interspecies chimerism capability of sheep iPSCs [54].

The analytical descriptions for each of the iPSCs discussed are detailed in Table 2.

**TABLE 2:** The history of iPSCs.

Cell type	Initiation cells	Reprogramming factor	Cultivation conditions	Characteristics	Reference
piPSC	Porcine fetal fibroblasts	Lentiviral transduction of hOCT4, hSOX2, hKLF4 and hc‐MYC	A culture medium standardised for human ESC containing 4 ng/mL human FGF2	Reprogramming efficiency ≈ 0.1%; positive for SSEA‐1; > P220	Derivation of induced pluripotent stem cells from pig somatic cells
Tibetan iPSC	Fibroblasts isolated from the Tibetan miniature pig	Transfected pMX plasmids containing epidermal growth factor protein or mouse Sox2, Klf4, Oct4 and c‐Myc	Dulbecco's modified Eagle's medium, high glucose with antibiotics, glutamine, pyruvate, NEAA, β‐mercaptoethanol, bFGF and FBS	The resulting iPS cell lines more closely resemble human ESC than cells from other species, have normal karyotype, stain positive for alkaline phosphatase, express high levels of ESC‐like markers (Nanog, Rex1, Lin28 and SSEA4), and can differentiate into teratomas composed of the three germ layers	Generation of induced pluripotent stem cell lines from Tibetan miniature pig
piPSC: VSMUi001‐D	Embryonic fibroblast cells	Using letroviral transduction approaches carrying hOCT4, hSOX2, hKLF4, hc‐MYC and hLIN28	Knock‐out DMEM, FBS, NEAA, glutamine, LIF, bFGF, β‐mercaptoethanol	Positive for alkaline phosphatase activity; expressed the pluripotency associated transcription factors including OCT4, SOX2, NANOG and surface markers SSEA‐1; exhibited a normal karyotype; formed embryoid bodies in vitro and teratomas in vivo	Generation of a pig induced pluripotent stem cell (piPSC) line from embryonic fibroblasts by incorporating LIN28 to the four transcriptional factor‐mediated reprogramming: VSMUi001‐D
piPSCs (pips m cells)	Pig embryonic fibroblasts	Lentiviral transduction of hOCT4, hSOX2, hKLF4 and hc‐MYC	LBX: Knock‐out DMEM, N2B27, FGF, hLIF, PD0325901, CHIR99021, SB431542, DOX	Expressed Oct4 and c‐Myc; both X chromosomes were active; formed embryoid bodies in vitro and teratomas in vivo	Efficient generation of mouse ESCs‐like pig induced pluripotent stem cells
3i/LAF‐iPSC	Pig embryonic fibroblast cells	Lentiviral transduction of hOCT4, hSOX2, hKLF4, hc‐MYC, Hbcl2l1	3i/LAF: GSK3βi (CHIR99021), SRCi (WH‐4‐023), WNTi (IWR‐1‐endo), LIF, Activin A and FGF2	Maintaining domed morphology; positive AP staining; expression of pluripotency genes; normal karyotypes; high passage numbers maintained genomic stability	Generation of stable integration‐free pig induced pluripotent stem cells under chemically defined culture condition
biPS cells	Bovine embryonic fibroblast cells.	Lentiviral transduction of bOCT4, bSOX2, bKLF4, bMYC, bLIN28, bNANOG	Knock‐out DMEM, L‐glutamine, NEAA, β‐mercaptoethanol, bFGF.	Normal karyotypes; positive AP staining; expression of pluripotency genes; teratomas in vivo	Generation of induced pluripotent stem cells from bovine embryonic fibroblast cells
Bovine iPSC line (biPS‐1)	Bovine fetal fibroblasts	By means of the PB transposition mediated gene transfer. (SOX2, OCT4, KLF4, c‐MYC, NANOG and LIN28)	F‐12 DMEM, knockout DMEM, l‐glutamine, NEAA, mercaptoethanol, penicillin, streptomycin, bFGF, hLIF	Normal karyotypes; positive AP staining; expression of pluripotency genes SSEA1, NANOG and SOX2; formed embryoid bodies in vitro and teratomas in vivo	Derivation and characterisation of bovine induced pluripotent stem cells by transposon mediated reprogramming
Bovine iPS	Primary bovine fetal fibroblasts were derived from E45 Qinchuan cattle embryos	By the introduction of piggyBac transposons with CAG promoting transcription factors (Oct3/4, Sox2, Klf4 and cMyc)	Knockout DMEM, FBS, L‐glutamine, NEAA, β‐mercaptoethanol, hLIF, bFGF	Reprogramming efficiency ≈ 40%; expression of pluripotency genes; not naïve pluripotent	Characterisation of the single‐cell derived bovine induced pluripotent stem cells
Bovine iPSCs	The bovine Sertoli cells isolated from the cryopreserved neonatal bull testis	Lentiviral transduction of hOCT4, hSOX2, hKLF4 and hc‐MYC	M3: knockout DMEM, P/S, NEAA, β‐mercaptoethanol, LIF, bFGF, PD 0325901	Normal karyotypes; positive AP staining; remain pluripotency in vitro/vivo after freezing/thawing	Transcriptome profile of bovine iPSCs derived from Sertoli cells
Bovine naïve‐like iPSCs	The primed bovine iPSCs		BEF feeder + naïve medium (NM). NM: knockout DMEM, P/S, NEAA, β‐mercaptoethanol, LIF, bFGF, PD0325901, CHIR99021, N2, recombinant human insulin, TGF‐β1, SB202190, SP600125, SB203580, Vitamin C	BEF feeder + NM enhance the conversion from the primed to naïve‐like bovine iPSCs	Naive‐like conversion of bovine induced pluripotent stem cells from Sertoli cells
Sheep iPS cells	The sheep ear primary fibroblasts	Lentiviral transduction of Oct4, Sox2, c‐Myc, Klf4, Nanog, Lin28, SV40 large T and hTERT	DMEM/F12, KnockOut DMEM, NEAA, L‐glutamine, β‐mercaptoethanol, DOX	Normal karyotypes; teratomas in vivo	Reprogramming of ovine adult fibroblasts to pluripotency via drug‐inducible expression of defined factors
Sheep iPSCs (siPSCs)	Sheep fibroblasts	Transfected pMX plasmids containing human Sox2, Klf4, Oct4 and c‐Myc	DMEM, FBS, insulin/transferrin/selenium, 2‐βmercaptoethanol, NEAA, glutamine, bFGF, mLIF	Normal karyotypes; expression of pluripotency genes OCT4 and SOX2; formed embryoid bodies in vitro and teratomas in vivo	Generation and characterisation of reprogrammed sheep induced pluripotent stem cells
Ovine‐induced pluripotent stem cells	Ovine embryonic fibroblasts	Transfected with murine cMyc, Klf4, Oct4 and Sox2	Human embryonic stem cell (ESC) medium	Expression of pluripotency genes NANOG; normal karyotypes; positive AP staining	Ovine‐induced pluripotent stem cells can contribute to chimeric lambs
bFGF/activin‐dependent ovine iPSCs	Ovine parental foetal fibroblasts	Lentiviral transduction of Oct4, Sox2, Klf4 and c‐Myc	Knock‐out DMEM, Glutamax, NEAA, M2‐mercaptoethanol, bFGF	These cells were basic fibroblast growth factor (bFGF)‐ and activin A‐dependent; positive AP staining; X chromosomal abnormalities; Not expressed endogenous NANOG	Ovine induced pluripotent stem cells are resistant to reprogramming after nuclear transfer
Sheep‐induced pluripotent stem cells (siPSCs)	Sheep kidney cells	Transfected by tetracycline (TET)‐on carrying Oct4, Sox2, c‐Myc, Klf4, Nanog, Lin28, hTERT, and SV40LT	DMEM/F12, KnockOut Serum Replacement, NEAA GlutaMAX, β‐mercaptoethanol, bFGF, DOX, vitamin C	Positive AP staining; expression of pluripotency genes; formed embryoid bodies in vitro	miR‐200c‐141 Enhances Sheep Kidney Cell Reprogramming into Pluripotent Cells by Targeting ZEB1
Sheep iPSCs	Fibroblasts of black‐bone sheep	Transfected with bovine OCT4, cMYC, SOX2, KLF4, porcine NANOG and human LIN28, SV40 large T antigen and human TERT	M15: knockout DMEM, FBS, P/S, GlutaMAX, NEAA, 2‐mercaptoethanol, LIF, vitamin C, DOX	Stable morphology, pluripotent marker expression, in vitro differentiation ability, and interspecies chimeric potential	Generation of sheep induced pluripotent stem cells with defined DOX‐inducible transcription factors via piggyBac transposition

## 6 Application Prospects of Livestock PSCs

**FIGURE 3:**
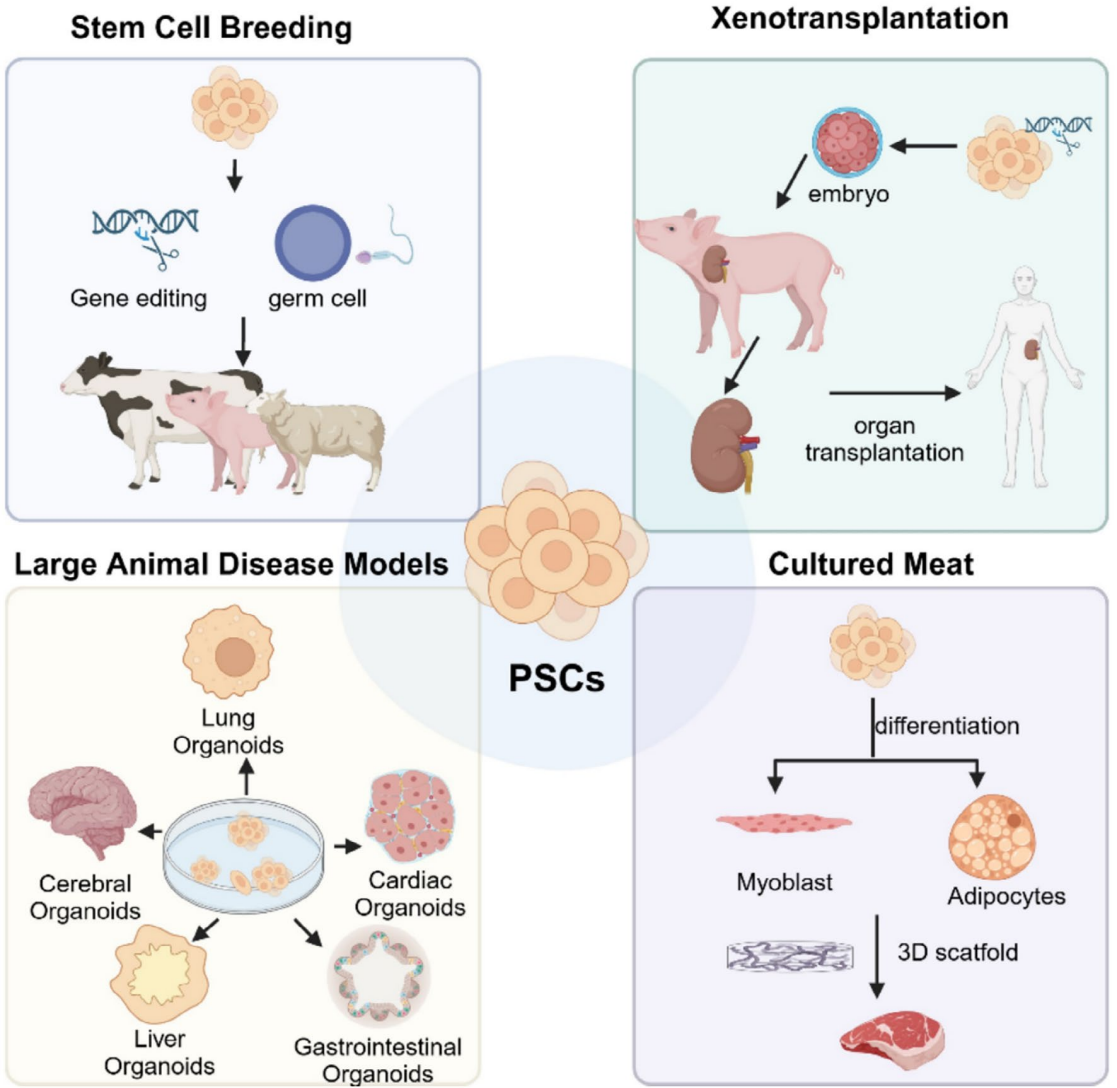
Application prospects of pluripotent stem cells in livestock. Livestock PSCs hold application prospects in stem cell breeding, large animal disease model creation, organ transplantation and cultured meat production. Figure was created using Biorender.com.

Livestock PSCs have the ability to self‐renew in vitro and possess multi‐directional differentiation capabilities, thus holding broad application prospects in stem cell breeding, large animal disease model creation, organ transplantation and cultured meat production (Figure 3).

### 6.1 Stem Cell Breeding

Biobreeding is a general term for modern agricultural biotechnology breeding, primarily utilising transgenic technology, gene editing, whole‐genome selection, synthetic biology, and other techniques to carry out efficient, precise, and targeted genetic improvement and variety cultivation in animals and plants. This technology has gradually become the mainstream approach in the field of livestock genetics and breeding, offering effective solutions for improving agricultural production efficiency and addressing global food security issues. PSCs have unlimited proliferation and self‐renewal capabilities, enabling multiple rounds of precise editing of multiple genes, thereby obtaining high‐yield, disease‐resistant, and high‐quality new livestock varieties. Additionally, PSCs can be directionally induced to differentiate into functional sperm and oocytes for the mass production of embryos in vitro. Livestock PSCs can be passaged indefinitely; however, during long‐term passaging, gene mutations may occur, which could affect the desired traits. Regular use of techniques such as whole‐genome sequencing can be employed to detect whether mutations have occurred. Moreover, although PGCs can be directionally induced into sperm and oocyte cells, the efficiency is quite low. A deeper understanding of the molecular mechanisms regulating germ cell development and optimisation of culture conditions may be necessary to improve the efficiency of inducing PSCs into germ cells. Do it this way, PSCs serve as a powerful material for biobreeding, offering new avenues for livestock breeding [55].

Combining stem cells with technologies such as genome sequencing and genome selection, offspring embryos can be obtained through in vitro germ cell induction and in vitro fertilisation, allowing for multi‐generation selection and mating in the laboratory, that is, stem cell breeding [56]. Compared to traditional breeding models, stem cell breeding enables direct intergenerational transfer of genetic information from parental embryos to offspring embryos, allowing for the reconstruction of the entire life cycle of mammals in vitro [57]. Stem cell breeding can rapidly achieve whole‐genome selection in vitro, increase selection intensity, shorten generation intervals, accelerate the breeding process, and cultivate new varieties with superior traits.

Genetic improvement is the foundation of livestock breeding, and stem cell breeding offers a novel approach to addressing issues such as genetic resource scarcity and variety degeneration. By combining stem cell technology with gene editing techniques such as CRISPR/Cas9 and base editors, multiple traits can be improved [58]. Furthermore, through embryo chimerism or in combination with nuclear transfer technology, a large number of livestock populations with improved productive traits can be rapidly obtained. Compared to traditional breeding techniques, stem cell breeding significantly accelerates the speed of livestock variety improvement and reduces breeding costs.

### 6.2 Application of Livestock PSCs in the Field of Xenotransplantation

Currently, many human diseases require organ transplantation for treatment. Each year, approximately 300,000 patients in China are waiting for organ transplants, but only about 6000 donated organs become available annually. Consequently, the severe shortage of donor organs significantly restricts the improvement of public health and the development of transplant medicine. However, xenotransplantation is one of the effective solutions to address the donor organ shortage. Pigs are evolutionarily closer to humans, their tissues do not contain human‐susceptible viruses, and they can be bred on a large scale, making pigs the best current source for human xenotransplantation organs [59]. With the advent of gene editing and stem cell technologies, significant progress has been made in pre‐clinical studies of xenotransplantation. For instance, using pig organs with knocked‐out α‐Gal and other immune‐related genes, such as porcine endogenous retrovirus‐related genes, can overcome the risks of immune rejection and cross‐species viral infections faced by xenotransplantation [60]. PSCs are one of the key materials for obtaining genetically modified pigs. Overexpression of two pro‐survival genes, MYCN and BCL2, in these cells can effectively prevent massive apoptosis of human cells in xenogeneic animals due to species differences. When these cells are injected into embryos of pigs with knockout SIX1 and SALL1 genes, which have defects in kidney development, they successfully integrate into the kidneys of 28‐day‐old pig foetuses, forming renal tubules and complete structures of human‐pig chimeric mid‐gestation kidneys. Although knocking out immune‐related genes and overexpressing survival genes can reduce immune rejection and increase the probability of successful transplantation, immune rejection remains a significant issue. Induced pluripotent stem cells (iPSCs) can be directed to differentiate into mesenchymal stem cells (MSCs), which possess stronger immune‐suppressive capabilities. These cells can secrete anti‐inflammatory factors such as TGF‐β1, HGF, IDO, PGE2 and IL‐6, inhibiting T cell proliferation and NK cell activity, thereby reducing immune rejection and minimising the host's immune response to the transplanted organ. Therefore, bioengineering techniques to recreate human organs suitable for transplantation within pigs, that is, xenochimera technology, provide possibilities for the preparation of functional human organs [61].

### 6.3 Application of Livestock PSCs in the Creation of Large Animal Disease Models

It is widely known that pigs are considered ideal model animals for human disease research due to their anatomical, physiological, and metabolic similarities to humans, as well as the similar mechanisms of disease development [34]. The complexity of diseases also makes multi‐gene modification a necessary technique for creating disease models. By modifying multiple genes simultaneously or sequentially, the genetic, pathological, and physiological processes of diseases can be more accurately reflected. Currently, somatic cell nuclear transfer is a common method for constructing models. However, the proliferative capacity of donor cells—porcine fibroblasts—is limited, making it difficult to obtain cloned pigs with multiple gene edits in one go. Re‐cloning and re‐isolating fibroblasts are required to achieve multiple rounds of gene editing, resulting in a lengthy and inefficient process. In contrast, PSCs offer the advantages of unlimited proliferation, self‐renewal, and the ability to withstand multiple consecutive gene editings, making them important materials for the rapid and efficient preparation of large animal disease models. However, most of the current gene editing tools are developed experimentally on fibroblasts, and there is a lack of tools specifically for livestock stem cells. When conducting multi‐gene modifications in livestock PSCs, not only is the editing efficiency low and the off‐target rate high, but the genetic modifications may also affect the cellular state. Therefore, it is necessary to validate the efficiency of gene editing tools on stem cells and to avoid off‐target effects to ensure the accuracy of disease models.

Organoid technology is a technique that utilises ESCs or PSCs to form tissue‐like structures in vitro through three‐dimensional culture, mimicking the structure and function of real organs. This provides a new model for disease research and clinical applications [62]. The team led by Hans Clevers pioneered this field by cultivating intestinal organoids from adult stem cells derived from mouse intestines, initiating the study of organoids [63]. Subsequently, heart organoids from human iPSCs and small intestinal organoids from mice were successfully cultivated [64]. In livestock, organoids are primarily used for human disease models, veterinary medicine, and nutrition research. For instance, 3D neural organoids established using pig ESCs have been utilised to study human central nervous system diseases [65]. Intestinal organoids cultivated from bovine intestinal epithelial stem cells have been used to investigate the infection mechanisms of pathogens such as Toxoplasma gondii and *Salmonella typhimurium* , providing valuable information for preventing the transmission of *Escherichia coli* from cattle to humans [66].

### 6.4 Application of Livestock PSCs in the Field of Cultured Meat

The rapid expansion and large‐scale development of the livestock industry have caused pollution and an impact on the ecological environment, water resources and land resources. For example, the massive emissions of greenhouse gases such as carbon dioxide (CO_2_), nitrous oxide (N_2_O), methane (CH_4_) and ammonia (NH_3_) severely harm the environment. Moreover, extensive cultivation of livestock occupies excessive land resources, leading to a scarcity of agricultural land [67]. Additionally, slaughtering inevitably causes suffering to animals, violating the requirements for farm animal welfare [68]. Cultured meat, which is produced by cultivating animal cells in vitro according to the growth patterns of meat in animal organisms, is expected to serve as an effective supplement to traditional livestock farming. It can also promote environmental sustainability and agricultural carbon reduction, making cultured meat a hot topic in the global future meat industry [69]. Stem cell technology is one of the key technologies for cultured meat production. By directing PSCs to differentiate into muscle cells and culturing them on 3D edible scaffolds, tissues similar to meat are created, making PSCs suitable as seed cells for cultured meat [70, 71]. The currently produced cell‐cultured meat utilises 3D printing technology to establish scaffold materials, which, although addressing the issue of a crumbly texture, has limited biodegradability and edibility. This problem can be solved by directing the differentiation of pluripotent stem cells into osteoblasts, which can better simulate real meat. Using fish fat stem cells and muscle stem cells, artificial fish meat has been successfully cultivated with a taste and texture indistinguishable from normal fish meat; however, the self‐renewal ability of muscle stem cells gradually weakens with each passage, preventing unlimited differentiation into muscle cells [72]. The team led by Jianyong Han utilised previously established, long‐term stable pgEpiSCs and animal‐free 3D scaffolds to successfully prepare cultured meat similar to normal meat indicators for the first time. This cultured meat has a distinct muscular appearance and texture, is rich in conditionally essential amino acids, and is less susceptible to contamination by foodborne pathogens, providing a theoretical and technical foundation for the industrialisation of cultured meat [72].

## 7 Conclusion

As the optimal materials for gene editing, embryonic stem cells (ESCs) and induced pluripotent stem cells (iPSCs) hold significant research value and broad application prospects in the fields of biological breeding, cell‐cultured meat, creation of medical large animal disease models, and regenerative medicine. While stable ESC and iPSC lines have been successfully established in mice and humans, progress in deriving livestock pluripotent stem cells has been slow, with true embryonic pluripotent stem cells and induced pluripotent stem cells yet to be achieved.

The maintenance of pluripotency and self‐renewal capacity in pluripotent stem cells typically requires specific culture medium components. For instance, mouse pluripotent stem cells are commonly cultured with CHIR99021, PD0325901 and LIF, whereas human pluripotent stem cells (hESCs) necessitate the additional supplementation of bFGF. However, the culture media optimised for mouse and human pluripotent stem cells are not directly applicable to livestock, likely due to species‐specific variations and interspecies differences. One notable distinction lies in the regulatory mechanisms of embryonic development across species. In mice, the embryonic development cycle is relatively short. By embryonic Day 4.5, during pre‐implantation development, the inner cell mass (ICM) rapidly proliferates and forms a cup‐shaped epiblast structure, facilitating implantation. Additionally, mouse blastocysts undergo developmental diapause. In contrast, livestock such as pigs exhibit a significantly prolonged period of pluripotency in the epiblast within the uterus, and during the pre‐implantation stage, a flat bilayer embryonic disc structure is formed, without experiencing developmental diapause.

To establish high‐quality livestock pluripotent stem cells capable of germline chimerism and transmission, future research should focus on the following key areas. First, a multi‐omics approach could be employed to better understand the molecular and cellular mechanisms underlying pluripotency in livestock species. Second, CRISPR whole‐genome knockout or activation libraries can be used to screen and identify key factors regulating livestock pluripotent stem cells. Third, small molecule library screening can be conducted to identify small molecule combinations suitable for maintaining the pluripotency of livestock pluripotent stem cells. Fourth, livestock autologous fibroblasts can be used as feeder layer cells. Studies have shown that bovine embryonic fibroblast (BEF) feeder layers perform better in maintaining the pluripotency and morphology of bovine iPSCs. In summary, a great deal of effort is still needed to establish truly livestock pluripotent stem cells, which have extensive and far‐reaching significance for agriculture and human medicine.

## Author Contributions

X.Z. conceived the review article. C.G. and W.Z. contributed to the initial drafting of the manuscript and overall editing. X.W. helped to modify the manuscript. D.Y., H.Z., and W.D. contributed to funding, overall supervision, and supported review development, overall editing, and critical overall manuscript revision.

## Conflicts of Interest

The authors declare no conflicts of interest.

## Data Availability

The data that support the findings of this study are available from the corresponding author upon reasonable request.
